# Heat Stress in Growing–Finishing Pigs: Effects of Low Protein with Increased Crystalline Amino Acids on Growth, Gut Health, Antioxidant Status and Microbiome

**DOI:** 10.3390/ani15060848

**Published:** 2025-03-15

**Authors:** Jihwan Lee, Sungwoo Park, Hyunju Park, Junseon Hong, Yongmin Kim, Yongdae Jeong, Soojin Sa, Yohan Choi, Joeun Kim

**Affiliations:** Swine Science Division, National Institute of Animal Science, Rural Development Administration, Cheonan 31000, Republic of Korea; junenet123@naver.com (J.L.); woht3013@naver.com (S.P.); guswn707@korea.kr (H.P.); gospel0342@korea.kr (J.H.); silveraz@korea.kr (Y.K.); yongdaejeong@korea.kr (Y.J.); soojinsa@korea.kr (S.S.)

**Keywords:** growing–finishing pigs, heat stress, low crude protein, crystalline amino acid, gut health

## Abstract

A total of sixty crossbred ([Landrace × Yorkshire] × Duroc) pigs with an initial body weight of 46.34 ± 0.13 kg were randomly assigned to four treatments under thermoneutral (TN, 22 °C) or heat-stress (HS, 31 °C) conditions for 54 d trial (Phase 1: 0–26 d; Phase 2: 27–54 d): a control diet (16% CP in phase 1, 14% CP in phase 2) under TN (PC), a control diet under HS (NC), a low crude protein (LCP) diet (14% CP in phase 1, 12% CP in phase 2) under HS (LCP) and an LCP diet with increased crystalline AA (an increase in 5% in Lys, Met, Thr and Trp based on calculated SID AA) (LCP5) under HS. Experimental treatments consisted of five replicate pens, with three pigs per pen. The results showed that HS reduced (*p* < 0.05) growth performance and nutrient digestibility compared to TN. However, LCP5 improved (*p* < 0.05) growth performance and nutrient digestibility compared to other HS groups. Heat stress adversely affected (*p* < 0.05) intestinal morphology, gut integrity and serum oxidative markers, but these effects were alleviated (*p* < 0.05) by LCP5 supplementation. Notably, LCP5 improved (*p* < 0.05) the production of butyric acids among short-chain fatty acid production and decreased (*p* < 0.05) *proteobacteria* and *Spirochaetota* in phylum in feces. These findings highlight the potential of LCP diets supplemented with crystalline AA as an effective nutritional strategy to mitigate the negative effects of HS on pigs, enhancing their performance, gut health and overall welfare in high-temperature environments.

## Simple Summary

Heat stress (HS) poses a significant challenge to the swine industry, impairing the growth performance, nutrient absorption, gut health and antioxidant capacity of growing–finishing pigs. This study investigated the effects of dietary strategies in mitigating the adverse effects of HS by formulating low crude protein diets supplemented with crystalline amino acids. The results revealed that this approach improved feed intake, growth, gut integrity and oxidative balance in pigs under HS conditions. Furthermore, it positively influenced the gut microbiota, promoting beneficial short-chain fatty acids while suppressing harmful microbial populations. These findings provide valuable insights into practical and sustainable nutritional solutions to enhance pig performance and welfare in high-temperature environments.

## 1. Introduction

Global surface temperatures have been continuously increasing due to anthropogenic climate change. According to NASA report [1], the Earth’s average surface temperature has risen by approximately 1.47 °C compared to the pre-industrial (1850–1900) average. This increase is particularly noteworthy as it coincides with an unprecedented record-breaking heat phenomenon that persisted for 15 consecutive months from June 2023 to August 2024. The rapid pace of climate change poses considerable risks to natural ecosystems. Pigs are particularly vulnerable to climate-induced stress due to their relatively small lung size compared to their body mass and the absence of functional sweat glands, which limits their ability to dissipate heat through panting [2]. According to Eastwood [3], pigs experience heat stress (HS) when the ambient temperature (AT) exceeds 30 °C, even when the relative humidity (RH) is below 50%. When exposed to HS, pigs experience heat stress, resulting in an approximately 2 °C increase in body temperature [4]. Heat stress negatively impacts animal growth, carcass quality, and reproductive performance, leading to significant economic losses [5,6]. Therefore, the development of nutritional strategies to mitigate the adverse effects of heat stress in pigs is necessary. One promising approach to addressing these issues is the use of low crude protein (LCP) diets supplemented with crystalline amino acids (AA). Compared to other nutrients, crude protein (CP) generates more heat during digestion, absorption, and metabolism, making it the primary contributor to heat production [7]. Additionally, during HS, voluntary feed intake decreases by 8% to as much as 48% to reduce heat production [8]. Previous studies have shown that the use of crystalline AA not only improves the efficiency of AA absorption but also minimizes digestive heat production as they exist in their simplest form and do not require enzymatic breakdown [9,10]. Kerr et al. [11] reported that LCP diets supplemented with crystalline AA reduce total heat production in growing pigs. Consequently, LCP diets with crystalline AA can mitigate the negative effects of additional heat stress caused by protein metabolism. Furthermore, supplying free AA, which are easily digestible and absorbable, can serve as a strategy to counteract the reduction in voluntary feed intake and the resulting decrease in performance. Additionally, under stress conditions, AA are utilized more for energy metabolism in the liver and the synthesis of heat shock proteins (HSP) rather than for muscle protein synthesis. Therefore, supplementation of additional AA can help compensate for the increased AA requirements during HS. Previous studies have also indicated that various stressors increase AA demands for energy metabolism and HSP synthesis in different animal species [12,13,14]. These studies suggest that additional AA supplementation can help alleviate heat stress; however, the optimal supplementation levels remain unclear. Moreover, some studies have reported that LCP diets did not affect growth performance compared to normal CP diets in pigs weighing less than 20 kg or more than 70 kg, and in some cases, even reduced average daily feed intake (ADFI) [15,16]. Therefore, this study hypothesizes that supplementing standardized ileal digestible (SID) Lys, Met, Thr and Trp at levels 5% higher than NRC requirements will further enhance the heat stress-mitigating effects of LCP diets. This 5% increase was selected based on the premise that additional AA would serve as a more readily available nutrient source under HS conditions, thereby improving gut health, feed intake and overall growth performance. Considering recent climate trends, where high temperatures persist even at night, this study specifically aimed to evaluate these effects under chronic HS conditions. The study was conducted to determine whether this level of amino acid supplementation could provide additional benefits under HS conditions compared to conventional CP and LCP diets.

## 2. Materials and Methods

### 2.1. Experimental Animal and Design

A total of 60 crossbred ([Landrace × Yorkshire] × Duroc) female pigs with an average body weight of 46.34 ± 0.13 kg were used in a 54 d trial, divided into two phases: phase 1 (0–26 d) and phase 2 (27–54 d). Pigs were randomly assigned to four treatments using a completely randomized block design based on their initial BW. Experimental treatments consisted of five replicate pens, with three pigs per pen. Each pen was equipped with a self-feeder and nipple drinker to allow ad libitum access to feed and water. This study was conducted in two environmental conditions. In the thermoneutral (TN) condition, the ambient temperature (AT) was maintained at 22 °C, while in the HS condition, the AT was set at 31 °C throughout the experiment. A control diet based on corn–soybean meal was formulated to meet and exceed the National Research Council nutrient requirements [17]. The crude protein (CP) levels were set at 16% and 14% in phases 1 and 2, respectively (Table 1). The treatments were as follows: the control diet (CP 16% and 14% in phase 1 and phase 2) under TN conditions (positive control, PC), control diet under heat stress (HS) conditions (negative control, NC), low crude protein (LCP) diet (CP 14% and 12% in phase 1 and phase 2) supplemented with crystalline AA under HS conditions (LCP) and LCP diet supplemented with increased crystalline AA (an increase in 5% in Lys, Met, Thr and Trp based on calculated SID AA) under HS conditions (LCP5).

### 2.2. Growth Performance

The pigs in each pen were weighed, and feed intake was recorded at initial, 26 d and final days (54 d of age) to calculate the average body weight (BW), average daily gain (ADG), daily feed intake (ADFI) and feed efficiency (G/F).

### 2.3. Nutrient Digestibility

Five days before fecal sample collection, 0.2% chromic oxide (Cr_2_O_3_) was formulated into the diets. At the end of each phase, fecal samples were collected via rectal massage from three pigs per pen to determine the apparent total tract digestibility (ATTD) of nutrients. The fecal collection was conducted at the end of each phase, immediately before body weight measurement. The collected samples were immediately transported to the laboratory and stored at −20 °C until further processing. Before analysis, the samples were dried in a digital hot air oven at 65 °C for 72 h. Subsequently, the dried samples were ground thoroughly and passed through a 1 mm sieve, and then ATTD of dry matter (DM), crude protein (CP) and gross energy (GE) were analyzed following the guidelines provided by AOAC [18]. Chromium concentration was measured using a UV-1201 spectrophotometer (Shimadzu, Kyoto, Japan). Approximately 2 g of feed and fecal samples were combusted in a Parr 6400 oxygen bomb calorimeter (Parr Instrument Co., Moline, IL, USA) to determine the gross energy of the samples. Nitrogen content was analyzed using a Tecator™ Kjeltec 8400 analyzer (Höganäs, Sweden). The ATTD of DM, CP and GE were calculated following the method described by Stein et al. [19].

### 2.4. Intestinal Morphology

At the end of the final phase, one pig per pen was euthanized prior to sample collection, and an approximately 3 cm segment of the duodenum, jejunum and ileum was collected. The collected intestinal sections were rinsed with phosphate-buffered saline and preserved in 10% formalin. The preserved tissues were then processed into paraffin blocks by sectioning them into smaller pieces. These paraffin-embedded blocks were sliced into 4 mm transverse sections using a Microtome (Microm HM340E, Thermo Fisher Scientific, Waltham, MA, USA) and, then, the collected sections were stained using hematoxylin and eosin and mounted on microscope slides. Villus height (VH), crypt depth (CD) and the ratio of villus height to crypt depth (VH/CD) were measured following the method described by Choi et al. [20]. Briefly, the stained sections were examined using a Vanox-S Microscope (Olympus Corporation, Lake Success, NY, USA). Image analysis was performed using SPOT basic imaging software (Version 5.6, Diagnostic Instruments, Sterling Heights, MI, USA).

### 2.5. Indirect Indicators of Gut Integrity in the Serum

At the end of each phase, 5 mL of the blood was collected via the jugular vein of one pig per pen using a sterilized syringe and stored in the K_3_EDTA evacuated tubes (Greiner Bio-One, Kramsmenster, Austria). The samples were centrifuged at 1500× *g* for 15 min at 4 °C. Serum samples were immediately transported to 1.5 mL plastic tubes and stored at −80 °C until analysis. Porcine zonulin (PZ; MBS2607498, MyBioSource, San Diego, CA, USA), Lipopolysaccharide (LPS; MBS269464, MyBioSource, San Diego, CA, USA) and porcine Lipopolysaccharide Binding Protein (PLBP; MBS074991, MyBioSource, San Diego, CA, USA) were analyzed by commercial assay kits.

### 2.6. Indicators of Inflammation and Oxidative Status in the Serum

Indicators of oxidative status were analyzed from the serum samples of one pig per pen stored at −80 °C. More specifically, tumor necrosis factor-α (TNF-α; MBS2019932, MyBioSource, San Diego, CA, USA), reactive oxygen species (ROS; MBS2602780, MyBioSource, San Diego, CA, USA), hydrogen peroxide (H_2_O_2_; MBS2540478, MyBioSource, San Diego, CA, USA), total antioxidant capacity (TAC; MBS9718973, MyBioSource, San Diego, CA, USA), superoxide dismutase (SOD; MBS265304, MyBioSource, San Diego, CA, USA), malondialdehyde (MDA; MBS268793, MyBioSource, San Diego, CA, USA) and catalase (MBS7615126, MyBioSource, San Diego, CA, USA) were measured by commercial assay kits.

### 2.7. Short Chain Fatty Acid (SCFA) Production in Feces

At the end of each phase, a fresh fecal sample was collected via rectal massage from three pigs per pen and then pooled and stored at −80 °C until analysis. SCFAs were measured following the method described by Oh et al. [21]. Briefly, approximately 1 g of feces was diluted with 2 mL of distilled water. The mixture was centrifuged at 10,000× *g* for 20 min at 4 °C to obtain a supernatant. Subsequently, 25% metaphosphoric acid solution was added to the supernatant in a 9:1 ratio and centrifuged again at 3000× *g* for 10 min. The supernatant was filtered through a 0.45 mm filter membrane after being aspirated with a syringe. SCFAs, including acetate, propionate, butyrate, valerate, isobutyrate, isovalerate and total SCFAs, were analyzed using a YL 6500 gas chromatograph (Anyang, Republic of Korea) equipped with a TRB-G43 capillary column (0.53 mm inner diameter, 30 m length and 3 μm film thickness) and a flame ionization detector. The column temperature was initially set to 70 °C and increased to 150 °C after 3 min. The injection volume was 1 μL, with both the injector and detector temperatures maintained at 250 °C.

### 2.8. Fecal Microbiome

This analysis was conducted based on the methods of previous studies, with slight modifications [22,23]. Deoxyribonucleic acid (DNA) was extracted from frozen fecal samples stored at −80 °C, intended for SCFA analysis, using the QIAamp^®^ DNA Stool Mini Kit (Qiagen GmbH, Hilden, Germany) following the manufacturer’s protocol. DNA quality and concentration were assessed with a NanoDrop 2000 spectrophotometer (Thermo Fisher Scientific, Waltham, MA, USA). For amplicon PCR, the 16S rRNA gene’s V3-V4 region was amplified using specific primers: V3 (forward): 5′-TCGTCGGCAGATGTGTATAGACAGCCTGCGNGGCWGCAG-3′ and V4 (reverse): 5′-GTCTCGGGGGGG GGTAGAGAGAGAGAGAGAGACHVGTATATCC-3′. The prepared library was then sent to Macrogen (Macrogen, Inc., Seoul, Republic of Korea) for sequencing using the MiSeq platform (Illumina, San Diego, CA, USA). For sequence processing, QIIME2 (version 2021.11) was used. Sequences were demultiplexed using the QIIME2 demux emp-paired function and denoised using the Deblur plugin. A phylogenetic tree of ASVs was constructed, and the taxonomy of each ASV was assigned using a pre-trained classifier with the reference SILVA database.

### 2.9. Statistical Analysis

Statistical analyses on all data in this study were performed using JMP 16.0 (SAS Institute Inc, Cary, NC, USA). Treatment means were separated using the Tukey Honestly Significant Difference (HSD) test. Statistical significance was considered when *p* < 0.05. MicrobiomeAnalyst tool (available at: http://www.microbiomeanalyst.ca, accessed on 7 January 2025) [24] was performed for analyzing α-diversity, which included calculations of the observed species, Chao1, ACE, Shannon and Simpson indices. The one-way Analysis of Variance (ANOVA) was used to estimate alpha diversity indexes based on Observed species, Chao1, Shannon and Simpson. Beta diversity community was represented via the Bray–Curtis index, Jaccard index, Unweight UniFrac Distance and Weight UniFrac Distance using a permutational multivariate ANOVA (PERMANOVA) statistics and was visualized with principal coordinate analysis (PCoA) plot.

## 3. Results

### 3.1. Growth Performance

The effects of an LCP diet supplemented with increased crystalline AA on growth performance in growing–finishing pigs during HS conditions are summarized in Table 2. Pigs exposed to HS (NC, LCP and LCP5) had significantly lower (*p* < 0.05) BW at 26 d and final BW than pigs under TN conditions (PC). Although the final BW of the LCP5 group was slightly higher than that of the NC and LCP groups, the difference was not statistically significant. Similarly, the HS groups had reduced (*p* < 0.05) ADG and G/F during phases 1, 2, and the overall period compared to the PC group. Additionally, there was a tendency (*p* = 0.0615) for HS to decrease ADFI over the entire study compared with the PC group.

### 3.2. Nutrient Digestibility

The effects of the LCP diet supplemented with increased crystalline AAs on nutrient digestibility in growing–finishing pigs during HS conditions are summarized in Table 3. The NC group had significantly lower (*p* < 0.05) ATTD of DM and CP in phases 1 and 2, except for GE than the PC group. LCP5 achieved similar ATTD of CP and GE levels compared to those of the PC group in phases 1 and 2.

### 3.3. Intestinal Morphology

The effects of an LCP diet supplemented with increased crystalline AA on intestinal morphology in growing–finishing pigs during HS conditions are summarized in Table 4. There was no significant difference (*p* > 0.05) in jejunal morphology and CD in the duodenum and ileum among treatments. However, the NC and LCP groups had decreased (*p* < 0.05) VH and VH/CD in the duodenum and ileum compared to the PC group. The LCP5 group achieved VH and VH/CD levels in the duodenum and ileum similar to those of the PC group.

### 3.4. Indirect Indicators of Gut Integrity

The effects of the LCP diet supplemented with increased crystalline AA on indirect indicators of gut integrity in growing–finishing pigs during HS conditions are summarized in Table 5. NC group had higher (*p* < 0.05) PZ, LPS and PLBP concentrations in the serum in phases 1 and 2 than the PC group. Similarly, the LCP and LCP5 groups had higher (*p* < 0.05) LPS concentration in the serum in phase 2 than the PC group. However, there was no significant difference (*p* > 0.05) in LPS concentration in the serum in phase 1 and PLBP concentration in the serum in phases 1 and 2 between the LCP groups and the PC group. Notably, the LCP5 group had the lowest (*p* < 0.05) PZ and PLBP concentration in the serum in phases 1 and 2 and also had, numerically, the lowest LPS concentration in the serum in phases 1 and 2 among pigs exposed to HS.

### 3.5. Indicators of Inflammation and Oxidative Status

The effects of the LCP diet supplemented with increased crystalline AA on indicators of inflammation and oxidative status in growing–finishing pigs during HS conditions are summarized in Table 6. There was no significant difference (*p* > 0.05) in ROS, H_2_O_2_, SOD, MDA and glutathione concentrations in the serum in phase 1, and TAC, MDA, catalase and glutathione concentrations in the serum in phase 2 among treatments. For an indicator of inflammation status, the NC group had higher (*p* < 0.05) TNF-α concentration in the serum in phases 1 and 2 than the PC group. For oxidative status, in phase 1, the NC and LCP groups had lower (*p* < 0.05) TAC and catalase concentrations in the serum than the PC groups. However, LCP5 achieved similar TAC and catalase concentrations in serum compared to the PC group. In phase 2, NC and LCP groups had the highest (*p* < 0.05) ROS and H_2_O_2_ concentration, and the lowest (*p* < 0.05) SOD concentration in the serum compared to the PC group. The TNF-α, ROS and H_2_O_2_ concentration in the serum were mitigated to levels similar to those of the PC group by supplementing LCP and increased crystalline AA. Notably, LCP5 achieved a SOD concentration in the serum similar to those of the PC group.

### 3.6. Short Chian Fatty Acid (SCFA) Production in Feces

The effects of the LCP diet supplemented with increased crystalline AA on short-chain fatty acids in the feces of growing–finishing pigs during HS conditions are summarized in Table 7. There was no significant difference (*p* > 0.05) in acetic acid, propionic acid, valeric acid and iso-butyric acid production in the feces in phases 1 and 2 among treatments. The NC group had lower (*p* < 0.05) butyric acid production in the feces in phases 1 and 2 compared to the PC group. However, there was no significant difference (*p* > 0.05) in butyric acid production and iso-valeric acid production in the feces between the LCP5 and PC groups.

### 3.7. Fecal Microbiome

**Figure 1:**
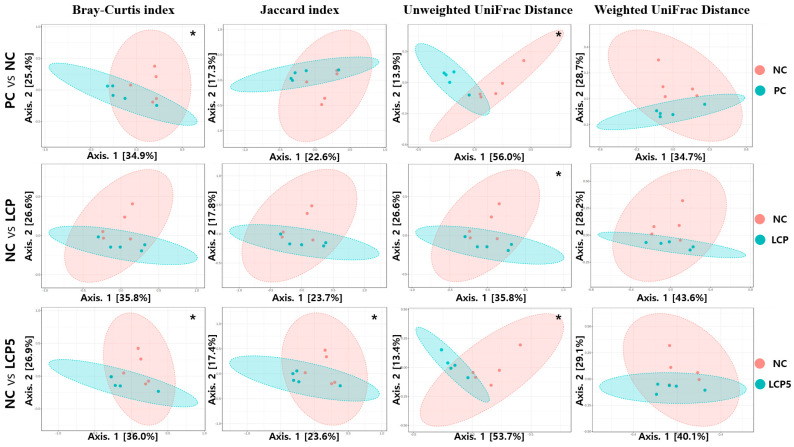
Effects of low protein with increased crystalline amino acids on beta diversity in feces in in growing–finishing pigs during heat stress. Statistical differences were assessed using permutational multivariate ANOVA (PERMANOVA). Abbreviation: PC, control diet (16% CP in phase 1, 14% CP in phase 2) under TN conditions (22 °C); NC, control diet (16% CP in phase 1, 14% CP in phase 2) under HS conditions (31 °C). LCP, low crude protein diet (14% CP in phase 1, 12% CP in phase 2) with crystalline amino acids under HS conditions (31 °C); LCP5: low crude protein diet (14% CP in phase 1, 12% CP in phase 2) with increased crystalline AA (an increase in 5% in Lys, Met, Thr and Trp based on calculated SID AA) under HS conditions (31 °C). *, *p* < 0.05.

The effects of an LCP diet supplemented with increased crystalline AAs on alpha diversity and the relative frequency of bacterial communities in feces in growing–finishing pigs during HS conditions are summarized in Table 8. The results of beta diversity PCoA plots are summarized in Figure 1. There was no significant difference (*p* > 0.05) in Shannon and Simpson indices in alpha diversity as well as in the relative frequencies of taxonomies at family and genus levels among treatments. The NC group had the lowest (*p* < 0.05) ACE and Chao 1 index in alpha diversity compared to the PC group. The ACE and Chao1 indices in the LCP and LCP5 groups were increased to levels comparable to those of the PC group. No significant differences (*p* > 0.05) in the relative frequencies of *Firmicutes*, *Bacteroidota*, *Actinobacteriota*, *Desulfobacterota* and *Euryarchaeota* at the phylum level were found among treatments. The NC group had higher (*p* < 0.05) relative frequencies of *Proteobacteria* and *Spirochaetota* compared to the PC group. The relative frequencies of *Proteobacteria* and *Spirochaetota* in the LCP and LCP5 groups were decreased to levels comparable to those of the PC group. There was no significant difference (*p* > 0.05) in the Jaccard index and Weight UniFrac Distance between the PC and NC groups. The Bray–Curtis index, Jaccard index and Weight UniFrac Distance among beta diversity indices in feces were not different (*p* > 0.05) between the NC and LCP groups. Weight UniFrac Distance among beta diversity indices in feces was not different (*p* > 0.05) between the NC and LCP5 groups. However, there were significant (*p* < 0.05) visible cluster separation effects in the Bray–Curtis index and Weighted UniFrac Distance between the PC and NC groups. Significant visible cluster separation effects in the Unweighted UniFrac Distance between the NC and LCP groups were observed (*p* < 0.05). Also, significant visible cluster separation effects in the Bray–Curtis index, Jaccard index and Unweighted UniFrac Distance between NC and LCP5 group were observed (*p* < 0.05).

## 4. Discussion

In this study, high ambient temperature caused HS to growing–finishing pigs, leading to decreased FI and weight gain. According to previous studies [25,26], there was a direct correlation between ambient temperature and FI since pigs can alter their FI to control heat production. Furthermore, Silva et al. [27] reported that FI could be reduced by approximately 40 g for every 1 °C that exceeds the TN zone. Moreover, Lan and Kim [28] showed that cyclic HS significantly decreased ADFI along with ADG and final BW compared to TN. Consistent with this, the FI of pigs exposed to HS except for LCP5 declined by 400 g as the temperature was increased by 9 °C in this study despite chronic HS conditions. Li and Patience [29] noted that proteins contribute the greatest caloric increase compared to carbohydrates and oils. Furthermore, Kerr et al. [30] reported that the availability of AA could be reduced by the reduction in FI. Thus, in this study, we implemented a supplementation of LCP and increased crystalline AA as a nutritional strategy to mitigate the negative effect of HS on FI and availability. To increase SID Lys, Met, Thr, and Trp levels by 5% above the NRC requirement [17], additional was provided. Wang et al. [31] explained that the main reason for the decline in performance under HS is due to the reduction in essential AA uptake. Also, free AAs could be absorbed without requiring enzymatic digestion and, thus, do not generate body heat during the digestive process [10]. Therefore, the improvement in growth performance observed with LCP and increased crystalline AA supplementation in this study could be attributed to a reduction in heat increment and the provision of adequate essential amino acids. Also, an additional 5% Trp supplementation to increase SID Trp levels in this study may contribute to the increased FI of pigs exposed to HS. Trp, as a precursor to serotonin, may regulate feed intake by boosting serotonin activity in the brain [32]. Additional Trp supplementation improved serotonin synthesis in the hypothalamus, which increased FI impaired by excess Leu [33]. This alleviation in FI and growth compromised by HS in this study might be explained by the above-mentioned functions of Trp. However, José Karpeggiane de Oliveira et al. [34] reported that LCP diets and extra AA supplementation did not influence growth performance under cyclic HS conditions. These differences may be due to the difference in pigs, weights, HS condition and experimental periods. The intestine is the main site of nutrient absorption as well as a barrier against abnormal presentation of luminal constituents and pathogens [35]. HS diverts blood flow to the periphery as part of thermoregulatory mechanisms to maximize radiant heat dissipation. This re-distribution of blood flow can reduce the oxygen supply to the intestine, leading to diminished oxygen availability in the intestinal tissues and initiating localized inflammation and damaging intestinal integrity [36,37,38,39]. In this study, the HS decreased VH and VH/CD, leading to compromising duodenal and ileal morphology. This finding was in agreement with previous studies which found that pigs exposed to AT above 35 °C had short villi of the small intestine [40,41,42]. González et al. [43] noted that shortened villi of the intestine may result in impaired digestive and absorptive functions, leading to lower nutrient availability for the growth of HS pigs. Moreover, Feng et al. [44] reported that VH/CD is comprehensively considered to reflect the intestinal absorptive capacity. Increased VH/CD serves greater efficiency in intestinal absorption. Consistent with this, we observed the lower ATTD of DM and CP in pigs exposed to HS in this study. The decreased nutrient digestibility observed in this study may be attributed to the reduced VH and VH/CD caused by HS. As expected, HS increased PZ, LPS, PLBP, pro-inflammatory cytokine (i.e., TNF-α) and the release of ROS and H_2_O_2_ in the serum, while decreasing TAC and antioxidant enzymes such as SOD and catalase compared to TN. Tight junction proteins consist of complex structures made up of different proteins and serve as a barrier to prevent the movement of large molecules [45]. Zonulin, as one of the tight junction proteins, is a regulator that influences intestinal permeability by controlling intracellular tight junctions, and circulating zonulin has been used as a potential indicator of intestinal permeability [46]. Thus, in this study, an increased zonulin concentration in the serum of pigs exposed to HS indicates increased permeability due to compromised gut integrity. In this study, the increased LPS, PLBP, and TNF-α concentration in serum may be attributed to increased permeability. Consistent with our findings, exposure to HS caused increased leakage of the intestine, which, ultimately, facilitated the translocation of endotoxin, commonly referred to as LPS, from the intestine into the bloodstream [47,48,49,50,51]. According to the study by Xia et al. [52], a systemic immune response was induced by pathogenic agents including endotoxin, antigens and bacteria entering the blood. Lian et al. [53] reported that increased ROS and a reduction in SOD can contribute to excessive oxidative stress. ROS is a primary consequence of HS, while SOD and catalase function as key antioxidant factors, and TAC reflects the host’s overall antioxidant capacity. In HS conditions, serum peroxidation significantly increases, rapidly depleting serum antioxidant factors and impairing the organism’s antioxidant capacity, ultimately resulting in oxidative stress within the body [54]. Increased H_2_O_2_ and ROS in this study could explain the activation of the above-mentioned serum peroxidation, and the above mechanism may lead to a reduction in antioxidant enzymes.

On the other hand, supplementation of LCP with increased crystalline AAs under HS conditions improved intestinal morphology and gut integrity along with nutrient digestibility to levels comparable to those of pigs in TN conditions. Trp, which was used in this study, is typically included in the regulation of the mammalian target of rapamycin (mTOR) which plays a role in maintaining intestinal barrier integrity [55,56]. Yan et al. [57] and Xie et al. [58] reported that the mTOR signaling pathway stimulated the proliferation of intestinal epithelial cells and reduced apoptosis, thereby maintaining the improvement of nutrient digestibility observed in this study may be attributed to the reduced VH and VH/CD caused by HS. Furthermore, supplementation of LCP diets with increased crystalline AA under HS conditions led to a reduction in the concentrations of PZ, LPS, and PLBP—indirect markers of intestinal integrity—as well as lower serum TNF-α levels. Additionally, SOD and catalase concentrations increased, while H_2_O_2_ levels decreased to values similar to those observed in pigs under TN conditions. These results suggest that additional crystalline AA supplementation helps mitigate the detrimental effects of HS by improving intestinal integrity and enhancing antioxidant capacity. Duarte et al. [59] reported that additional Trp supplementation to a low CP diet increased the expression of tight junction protein on the intestinal mucosa. Furthermore, an in vitro study showed that Trp promoted the expression of tight junction proteins in porcine small intestinal epithelial cells [60]. In this study, the reduction in PZ, LPS and PLBP concentrations in the serum of pigs fed LCP diets with increased crystalline AAs, especially Trp, could support the above-mentioned effects of Trp on tight junction proteins. Also, the reduction in the TNF-α concentration in the serum of pigs in the LCP5 group may be attributed to the alleviation of tight junction protein by Trp. However, a high dosage of Trp reduced the abundance of tight junction protein such as zonula occludens-1 and occludin in weaning pigs [61]. According to Li et al. [62], excessive Trp can induce cytotoxicity in cells. This discrepancy may be attributed to differences in the dosage of Trp used in various studies. Furthermore, the improvements in gut health and antioxidant capacity observed in LCP5 group may also be explained by the supplementation of Thr and Met. To increase SID Thr and Met levels by 5% relative to NRC requirements, we supplemented the diets with additional Thr and Met along with Trp. Previous studies have reported that supplementation of Thr can increase the abundance of β-defensins, a major class of host defense peptides in porcine intestinal epithelial cells, and similarly regulate the mTOR signaling pathway [63,64]. Additionally, increased AA supplementation, including Met, has been shown to reduce the LBP concentration in the serum during HS [65]. These findings suggest that the beneficial effects of the LCP5 diet on intestinal integrity and antioxidant capacity may be due to the combined effects of Trp, Thr and Met supplementation. Rehman et al. [66] reported that Met contributes to mitigating the negative effects of free radicals, ultimately improving antioxidant capacity both directly and indirectly. Met is widely recognized as one of the most oxidation-prone residues in proteins, and its supplementation has been shown to improve antioxidant activity in broilers exposed to HS [67,68]. Thus, this nutritional strategy, which includes additional AAs, such as Trp, Thr and Met, may contribute to improving gut health, immunity and antioxidant capacity in pigs under HS conditions.

The gut microbiota composition can significantly affect pig health and may be influenced by nutrients such as AAs and environmental factors [69,70]. Alpha diversity indices (i.e., ACE and Chao1) reflect the richness and diversity of fecal microbiota. In this study, pigs in the NC group exhibited lower alpha diversity indices, specifically ACE and Chao1, compared to other treatment groups. This suggests that HS negatively affected gut microbial diversity, which could have implications for gut health and overall physiological function. Consistent with our findings, previous studies have reported a decrease in ACE and Chao1 indices in animals exposed to HS [71]. Additionally, in this study, HS increased the population of *Proteobacteria* and *Spirochaetota* in phylum levels. According to a study by Grosu et al. [72], *Spirochaetota* are closely associated with gut dysbiosis and can produce specific byproducts that stimulate the intestinal lining, leading to inflammation and increased intestinal permeability. At the phylum level, *Proteobacteria* include species, such as *Salmonella*, *Shigella* and *Escherichia*, which are known to produce significant toxins [73,74,75]. However, He et al. [76] reported that HS did not affect ACE and Chao1 indices as well as Shannon and Simpson indices, which reflect the evenness of fecal microbiota. In contrast, another study have observed an increase in Chao1 index in pigs under HS conditions [77]. The effects of HS on the gut microbiota of pigs are complex as well as dynamic. In fact, Khosravi [78] emphasized that the alteration of the microbiota can vary depending on the temperature, duration and intensity adjusted to induce HS. However, the supplementation of LCP with increased AAs under HS conditions increased the ACE and Chao 1 indices among alpha diversities and decreased the abundance of *Spirochaetota* and *Proteobacteria* in the phylum. Also, this nutritional strategy showed significant visible cluster separation effects in beta diversity indices, such as the Bray–Curtis index, Jaccard index and Weighted UniFrac Distance, compared to the LC group. These improvements in this study may be elucidated by SCFA productions. This nutritional strategy increased the production of butyric acids, which had decreased during the high-temperature period, and decreased the production of iso-valeric acids, which had increased, bringing them to levels comparable to those in the TN pigs. Butyric acids serve as an energy source for the intestinal epithelium, play a role in immune system regulation and influence metabolic processes, and their deficiency may lead to the development of diseases by facilitating the growth of enteric pathogens [79]. Additionally, branched-chain fatty acids (BCFAs), such as iso-valeric acids, which are derived from the fermentation of amino acids, serve as a critical factor in the inducement of diarrhea in pigs [80]. Thus, this nutritional strategy may mitigate the negative effects of HS by providing sufficient nutrients in the form of free AAs, which can be directly absorbed without undergoing complex digestive processes, even in the intestine, where intestinal absorption is impaired due to HS. Additionally, the supplementation of Met, Thr and Trp used in this study may mitigate the negative effects of HS by positively supporting gut health, immunity and antioxidant capacity, ultimately preventing a dramatic decline in growth and nutritional absorption.

## 5. Conclusions

This study demonstrated that HS adversely affects growth performance, nutrient digestibility, gut integrity, antioxidant capacity and fecal microbiota in growing–finishing pigs. The supplementation of LCP diets with increased crystalline AAs partially mitigated these effects, showing numerical improvement in feed intake, growth and gut health, while significantly reducing oxidative stress markers. Additionally, it positively influenced the gut microbiota by increasing the amount of beneficial SCFAs and reducing harmful microbial populations. These findings highlight the potential of targeted dietary strategies to alleviate HS impacts, offering practical solutions for enhancing pig performance and welfare in high-temperature environments.

## Figures and Tables

**Table 1:** Ingredients and composition of experimental diets (as fed basis).

Items	Phase 1 (0–26 d)			Phase 2 (27–54 d)		
	PC/NC	LCP	LCP5	PC/NC	LCP	LCP5
Corn	68.77	73.71	73.57	74.43	79.47	79.36
Soybean meal, CP 44%	25.75	20.40	20.43	20.29	14.94	14.95
Beef Tallow	0.35	0.37	0.35	0.23	0.20	0.17
Molasses	3.00	3.00	3.00	3.00	3.00	3.00
L-Lysine, 78.8%	0.03	0.20	0.25	0.05	0.22	0.28
DL-Methionine, 98%	-	0.05	0.08	-	0.05	0.08
Threonine, 99%	-	0.04	0.07	-	0.05	0.08
Tryptophan, 99%	-	-	0.01	-	-	0.01
Limestone	0.70	0.68	0.68	0.70	0.67	0.67
Di-calcium phosphate	0.70	0.85	0.85	0.60	0.70	0.70
Salt	0.30	0.30	0.30	0.30	0.30	0.30
Choline	0.05	0.05	0.05	0.05	0.05	0.05
Mineral premix ^1^	0.15	0.15	0.15	0.15	0.15	0.15
Vitamin premix ^2^	0.15	0.15	0.15	0.15	0.15	0.15
Phytase	0.05	0.05	0.05	0.05	0.05	0.05
Total	100	100	100	100	100	100
Calculated value						
ME, kcal/kg	3300	3301	3301	3300	3300	3300
CP, %	16.00	14.00	14.00	14.00	12.00	12.00
SID Lys, %	0.85	0.85	0.89	0.73	0.73	0.77
SID Met, %	0.26	0.26	0.28	0.23	0.23	0.24
SID TSAA, %	0.52	0.52	0.55	0.47	0.47	0.49
SID Thr, %	0.55	0.55	0.57	0.48	0.48	0.49
SID Trp, %	0.18	0.18	0.18	0.15	0.15	0.16
Met: Lys	0.31	0.31	0.32	0.32	0.32	0.32
TSAA: Lys	0.61	0.61	0.61	0.64	0.64	0.64
Thr: Lys	0.65	0.65	0.64	0.66	0.66	0.64
Trp: Lys	0.21	0.21	0.20	0.21	0.21	0.21
Ca	0.59	0.59	0.59	0.54	0.54	0.52
Total P	0.52	0.52	0.52	0.47	0.47	0.46

Abbreviation: PC, control diet (16% CP in phase 1, 14% CP in phase 2) under TN conditions (22 °C); NC, control diet (16% CP in phase 1, 14% CP in phase 2) under HS conditions (31 °C); LCP, low crude protein diet (14% CP in phase 1, 12% CP in phase 2) with crystalline amino acids under HS conditions (31 °C); LCP5: low crude protein diet (14% CP in phase 1, 12% CP in phase 2) with increased crystalline AA (an increase in 5% in Lys, Met, Thr and Trp based on calculated SID AA) under HS conditions (31 °C); ME, metabolizable energy; CP, crude protein; SID, standardized ileal digestible; Lys, lysine; Met, methionine; TSAA, total sulfur amino acids; Thr, Threonine; Trp, Tryptophan; Ca, calcium; P, phosphorus. ^1^ Supplied per kilogram of diet: 16,000 IU vitamin A, 3000 IU vitamin D3, 5.0 mg vitamin B1, 20 mg vitamin B2, 4 mg vitamin B6, 0.08 mg vitamin B12, 40 IU vitamin E, 5.0 mg vitamin K3, 75 mg niacin, 40 mg pantothenic acid, 0.15 mg biotin, 0.65 mg folic acid. ^2^ Supplied per kilogram of diet: 0.25 mg Co, 50 mg Cu, 15 mg Mn, 25 mg Zn, 45 mg Fe, 0.35 mg I, 0.13 mg Se.

**Table 2:** Effects of low protein with increased crystalline amino acids on growth performance in growing–finishing pigs during heat-stress conditions.

Items	PC	NC	LCP	LCP5	SEM	*p*-Value
BW, kg						
Initial	46.38	46.20	46.26	46.50	1.643	0.9992
26 d	74.96 ^a^	66.08 ^b^	65.24 ^b^	66.32 ^b^	2.471	0.0432
Final	100.24 ^a^	83.04 ^b^	83.80 ^b^	87.46 ^b^	2.769	0.0015
Phase 1 (0–26 d)						
ADG, kg	1.10 ^a^	0.76 ^b^	0.73 ^b^	0.76 ^b^	0.050	0.0002
ADFI, kg	2.35	2.05	2.19	2.09	0.116	0.2950
G/F	0.48 ^a^	0.37 ^b^	0.33 ^b^	0.37 ^b^	0.025	0.0066
Phase 2 (27–54 d)						
ADG, kg	0.94 ^a^	0.63 ^b^	0.69 ^ab^	0.78 ^ab^	0.063	0.0171
ADFI, kg	2.95 ^a^	2.46 ^ab^	2.28 ^b^	2.81 ^ab^	0.135	0.0106
G/F	0.32	0.25	0.30	0.28	0.021	0.1622
Overall						
ADG, kg	1.02 ^a^	0.70 ^b^	0.71 ^b^	0.77 ^b^	0.041	0.0001
ADFI, kg	2.65	2.26	2.24	2.46	0.113	0.0615
G/F	0.39 ^a^	0.31 ^b^	0.32 ^b^	0.31 ^b^	0.015	0.0054

Abbreviation: PC, control diet (16% CP in phase 1, 14% CP in phase 2) under TN conditions (22 °C); NC, control diet (16% CP in phase 1, 14% CP in phase 2) under HS conditions (31 °C); LCP, low crude protein diet (14% CP in phase 1, 12% CP in phase 2) with crystalline amino acids under HS conditions (31 °C); LCP5: low crude protein diet (14% CP in phase 1, 12% CP in phase 2) with increased crystalline AA (an increase in 5% in Lys, Met, Thr and Trp based on calculated SID AA) under HS conditions (31 °C); BW, body weight; ADG, average daily gain; ADFI, average daily feed intake; G/F, feed efficiency; SEM, pooled standard error of the means. ^a,b^ Values within a row not sharing a superscript are significantly different at *p* < 0.05.

**Table 3:** Effects of low protein with increased crystalline amino acids on apparent total tract digestibility (ATTD) of nutrient in growing–finishing pigs during heat-stress conditions.

Items, %	PC	NC	LCP	LCP5	SEM	*p*-Value
Phase 1						
DM	85.81 ^a^	83.19 ^bc^	82.42 ^c^	83.71 ^b^	0.199	<0.0001
CP	82.26 ^a^	78.84 ^c^	80.54 ^b^	80.87 ^ab^	0.395	0.0002
GE	83.68 ^a^	83.57 ^a^	82.59 ^b^	83.47 ^ab^	0.234	0.0171
Phase 2						
DM	84.94 ^a^	82.40 ^b^	81.36 ^b^	81.64 ^b^	0.302	<0.0001
CP	79.39 ^a^	76.65 ^b^	77.81 ^ab^	78.19 ^ab^	0.606	0.0409
GE	81.95 ^a^	81.19 ^ab^	80.52 ^b^	81.16 ^ab^	0.266	0.0146

Abbreviation: PC, control diet (16% CP in phase 1, 14% CP in phase 2) under TN conditions (22 °C); NC, control diet (16% CP in phase 1, 14% CP in phase 2) under HS conditions (31 °C); LCP, low crude protein diet (14% CP in phase 1, 12% CP in phase 2) with crystalline amino acids under HS conditions (31 °C); LCP5: low crude protein diet (14% CP in phase 1, 12% CP in phase 2) with increased crystalline AA (an increase in 5% in Lys, Met, Thr and Trp based on calculated SID AA) under HS conditions (31 °C); DM, dry matter; CP, crude protein; GE, gross energy; SEM, pooled standard error of the means. ^a,b,c^ Values within a row not sharing a superscript are significantly different at *p* < 0.05.

**Table 4:** Effects of low protein with increased crystalline amino acids on intestinal morphology in growing–finishing pigs during heat-stress conditions.

Items	PC	NC	LCP	LCP5	SEM	*p*-Value
Duodenum						
VH, μm	518.80 ^a^	449.40 ^c^	497.00 ^b^	502.20 ^ab^	4.605	<0.0001
CD, μm	308.20	321.40	323.40	317.60	7.694	0.5285
VH/CD	1.68 ^a^	1.43 ^b^	1.54 ^ab^	1.59 ^a^	0.037	0.0016
Jejunum						
VH, μm	552.40	521.40	525.00	527.60	9.489	0.1269
CD, μm	395.60	398.20	404.40	380.60	9.822	0.3947
VH/CD	1.40	1.31	1.30	1.39	0.039	0.1862
Ileum						
VH, μm	458.80 ^a^	426.80 ^b^	429.20 ^b^	440.60 ^ab^	5.914	0.0057
CD, μm	257.60	276.80	271.60	257.20	7.635	0.2089
VH/CD	1.78 ^a^	1.55 ^b^	1.59 ^b^	1.72 ^ab^	0.048	0.0099

Abbreviation: PC, control diet (16% CP in phase 1, 14% CP in phase 2) under TN conditions (22 °C); NC, control diet (16% CP in phase 1, 14% CP in phase 2) under HS conditions (31 °C); LCP, low crude protein diet (14% CP in phase 1, 12% CP in phase 2) with crystalline amino acids under HS conditions (31 °C); LCP5: low crude protein diet (14% CP in phase 1, 12% CP in phase 2) with increased crystalline AA (an increase in 5% in Lys, Met, Thr and Trp based on calculated SID AA) under HS conditions (31 °C); VH, villus height; CD, crypt depth; VH/CD, villus height to crypt depth ratio; SEM, pooled standard error of the means. ^a,b,c^ Values within a row not sharing a superscript are significantly different at *p* < 0.05.

**Table 5:** Effects of low protein with increased crystalline amino acids on indirect indicators of gut integrity in the serum in growing–finishing pigs during heat-stress conditions.

Items	PC	NC	LCP	LCP5	SEM	*p*-Value
Phase 1						
PZ, ng/ml	11.75 ^c^	14.41 ^a^	13.98 ^ab^	13.39 ^b^	0.183	<0.0001
LPS, ng/ml	4.98 ^b^	6.55 ^a^	6.57 ^ab^	5.86 ^ab^	0.388	0.0460
PLBP, ng/ml	4.20 ^b^	6.54 ^a^	5.64 ^ab^	5.04 ^b^	0.486	0.0417
Phase 2						
PZ, ng/ml	11.34 ^c^	14.74 ^a^	14.22 ^ab^	13.70 ^b^	0.214	<0.0001
LPS, ng/ml	6.70 ^b^	9.35 ^a^	8.14 ^a^	8.01 ^a^	0.595	0.0425
PLBP, ng/ml	4.37 ^b^	5.85 ^a^	5.22 ^ab^	4.47 ^b^	0.367	0.0410

Abbreviation: PC, control diet (16% CP in phase 1, 14% CP in phase 2) under TN conditions (22 °C); NC, control diet (16% CP in phase 1, 14% CP in phase 2) under HS conditions (31 °C). LCP, low crude protein diet (14% CP in phase 1, 12% CP in phase 2) with crystalline amino acids under HS conditions (31 °C); LCP5: low crude protein diet (14% CP in phase 1, 12% CP in phase 2) with increased crystalline AA (an increase in 5% in Lys, Met, Thr and Trp based on calculated SID AA) under HS conditions (31 °C); PZ, porcine zonulin; LPS, lipopolysaccharide; PLBP, porcine lipopolysaccharide binding protein; SEM, pooled standard error of the means. ^a,b,c^ Values within a row not sharing a superscript are significantly different at *p* < 0.05.

**Table 6:** Effects of low protein with increased crystalline amino acids on indicators of inflammation and oxidative status in the serum in growing–finishing pigs during heat-stress conditions.

Items	PC	NC	LCP	LCP5	SEM	*p*-Value
Phase 1						
Inflammation status						
TNF-α, pg/mL	119.13 ^b^	165.69 ^a^	154.32 ^ab^	145.26 ^ab^	9.418	0.0189
Oxidative status						
ROS, U/mL	0.25	0.46	0.33	0.34	0.045	0.0789
H_2_O_2_, mmol/L	0.52	0.67	0.59	0.51	0.066	0.0652
TAC, mM	1.16 ^a^	0.97 ^b^	0.98 ^b^	1.01 ^ab^	0.039	0.0091
SOD, U/mL	10.49	9.16	10.00	10.19	0.499	0.3060
MDA, ng/mL	6.05	6.23	5.34	5.44	0.231	0.3984
Catalase, U/mL	6.28 ^a^	5.51 ^b^	6.09 ^b^	6.12 ^ab^	0.889	0.0311
Glutathione, ng/mL	0.47	0.46	0.48	0.51	0.074	0.9611
Phase 2						
Inflammation status						
TNF-α, pg/mL	136.40 ^b^	172.57 ^a^	155.24 ^ab^	142.68 ^b^	9.164	0.0599
Oxidative status						
ROS, U/mL	0.45 ^b^	0.65 ^a^	0.56 ^ab^	0.50 ^b^	0.099	0.0288
H_2_O_2_, mmol/L	0.40 ^b^	0.64 ^a^	0.52 ^b^	0.44 ^b^	0.013	0.0048
TAC, mM	1.12	1.05	1.08	1.10	0.028	0.2896
SOD, U/mL	10.94 ^a^	9.15 ^b^	9.33 ^b^	10.11 ^ab^	0.314	0.0272
MDA, ng/mL	5.90	7.25	6.59	6.18	0.846	0.0701
Catalase, U/mL	5.56	4.58	5.27	5.44	1.268	0.9471
Glutathione, ng/mL	0.49	0.50	0.50	0.52	0.142	0.9995

Abbreviation: PC, control diet (16% CP in phase 1, 14% CP in phase 2) under TN conditions (22 °C); NC, control diet (16% CP in phase 1, 14% CP in phase 2) under HS conditions (31 °C). LCP, low crude protein diet (14% CP in phase 1, 12% CP in phase 2) with crystalline amino acids under HS conditions (31 °C); LCP5: low crude protein diet (14% CP in phase 1, 12% CP in phase 2) with increased crystalline AA (an increase in 5% in Lys, Met, Thr and Trp based on calculated SID AA) under HS conditions (31 °C); TNF-α, tumor necrosis factor alpha; ROS, reactive oxygen species; H_2_O_2_, hydrogen peroxide; TAC, total antioxidant capacity; SOD, superoxide dismutase; MDA, malondialdehyde; SEM, pooled standard error of the means. ^a,b^ Values within a row not sharing a superscript are significantly different at *p* < 0.05.

**Table 7:** Effects of low protein with increased crystalline amino acids on short-chain fatty acids production in feces in growing–finishing pigs during heat-stress conditions.

Items, μmol/g	PC	NC	LCP	LCP5	SEM	*p*-Value
Phase 1						
Acetic acid	60.10	57.93	59.10	62.80	1.422	0.1361
Propionic acid	14.78	13.70	13.61	14.25	0.635	0.9915
Butyric acid	7.05 ^a^	5.54 ^b^	6.14 ^b^	6.68 ^ab^	0.267	0.0265
Valeric aicd	2.38	3.00	2.63	2.79	0.208	0.2363
Iso-butyric acid	3.14	3.53	2.92	2.94	0.158	0.0524
Iso-valeric acid	4.06 ^ab^	4.28 ^a^	3.80 ^b^	3.78 ^b^	0.113	0.0283
Phase 2						
Acetic acid	60.95	57.00	60.15	59.36	1.978	0.5420
Propionic acid	13.41	12.24	12.74	13.91	0.747	0.4328
Butyric acid	6.55 ^a^	5.56 ^b^	5.85 ^ab^	6.03 ^ab^	0.422	0.0317
Valeric aicd	2.65	2.98	2.85	2.72	0.203	0.6868
Iso-butyric acid	3.08	3.52	2.96	2.58	0.276	0.0571
Iso-valeric acid	4.13 ^ab^	4.43 ^a^	3.94 ^b^	3.84 ^b^	0.116	0.0141

Abbreviation: PC, control diet (16% CP in phase 1, 14% CP in phase 2) under TN conditions (22 °C); NC, control diet (16% CP in phase 1, 14% CP in phase 2) under HS conditions (31 °C). LCP, low crude protein diet (14% CP in phase 1, 12% CP in phase 2) with crystalline amino acids under HS conditions (31 °C); LCP5: low crude protein diet (14% CP in phase 1, 12% CP in phase 2) with increased crystalline AA (an increase in 5% in Lys, Met, Thr and Trp based on calculated SID AA) under HS conditions (31 °C); SEM, standard error of the means. ^a,b^ Values within a row not sharing a superscript are significantly different at *p* < 0.05.

**Table 8:** Effects of low protein with increased crystalline amino acids on alpha diversity and relative frequency of bacterial communities in feces in growing–finishing pigs during heat-stress conditions.

Items	PC	NC	LCP	LCP5	SEM	*p*-Value
Alpha-diversity						
ACE	225.64 ^a^	159.67 ^b^	225.26 ^a^	249.99 ^a^	14.710	0.0033
Chao1	226.08 ^a^	160.16 ^b^	224.25 ^a^	252.81 ^a^	15.200	0.0039
Shannon	3.25	3.07	3.19	3.27	0.161	0.8295
Simpson	0.89	0.89	0.89	0.88	0.024	0.9703
Relative frequency, %						
Phylum level						
*Firmicutes*	88.47	84.74	89.82	86.56	3.035	0.8990
*Bacteroidota*	9.28	10.91	7.68	10.46	2.357	0.4678
*Actinobacteriota*	0.49	0.37	0.55	1.18	2.362	0.1313
*Desulfobacterota*	0.35	0.50	0.37	0.35	0.100	0.7460
*Proteobacteria*	0.20 ^b^	1.09 ^a^	0.46 ^ab^	0.17 ^b^	0.167	0.0042
*Euryarchaeota*	0.07	0.26	0.13	0.15	0.062	0.2345
*Spirochaetota*	0.02 ^b^	1.97 ^a^	0.84 ^ab^	0.25 ^ab^	0.468	0.0400
Family level						
*Lactobacillaceae*	19.91	19.92	20.08	18.49	7.091	0.9983
*Clostridiaceae*	20.14	28.20	19.86	31.10	4.980	0.4254
*Erysipelotrichaceae*	9.37	8.54	9.34	7.52	1.351	0.7427
*Peptostreptococcaceae*	7.60	9.01	9.17	9.66	1.659	0.8356
*Selenomonadaceae*	2.00	5.52	4.69	5.34	1.561	0.3785
Genus level						
*Lactobacillus*	19.91	18.48	19.92	20.08	7.091	0.9983
*Clostridium_sensu_stricto_1*	20.09	28.04	29.72	30.92	4.959	0.4314
*Terrisporobacter*	3.39	7.00	5.65	5.53	1.379	0.3520
*Turicibacter*	8.62	8.35	9.09	7.06	1.416	0.7712

Abbreviation: PC, control diet (16% CP in phase 1, 14% CP in phase 2) under thermoneutral conditions (22 °C); NC, control diet (16% CP in phase 1, 14% CP in phase 2) under heat-stress conditions (31 °C). LCP, low crude protein diet (14% CP in phase 1, 12% CP in phase 2) with crystalline amino acids under heat-stress conditions (31 °C); LCP5: low crude protein diet (14% CP in phase 1, 12% CP in phase 2) with increased crystalline AA (an increase in 5% in Lys, Met, Thr and Trp based on calculated SID AA) under heat-stress conditions (31 °C); SEM, pooled standard error of the means. ^a,b^ Values within a row not sharing a superscript are significantly different at *p* < 0.05.

## Data Availability

The raw data supporting the conclusions of this study will be made available by the authors upon request.

## Abbreviations

The following abbreviations are used in this manuscript:

AA	Amino acid
ADFI	Average daily feed intake
ADG	Average daily gain
ANOVA	Analysis of variance
AT	Ambient temperature
ATTD	Apparent total tract digestibility
BCFA	Branched chain fatty acid
BW	Body weight
Ca	Calcium
CD	Crypt depth
CP	Crude protein
Cr_2_O_3_	Chromic oxide
DM	Dry matter
DNA	Deoxyribonucleic acid
G/F	Feed efficiency
GE	Gross energy
H_2_O_2_	Hydrogen peroxide
HSD	Honestly significant difference
HSP	Heat shock protein
IPCC	Intergovernmental Panel on Climate Change
LCP	Low crude protein
Leu	Leucine
LPS	Lipopolysaccharide
Lys	Lysine
MDA	Malondialdehyde
ME	Metabolizable energy
Met	Methionine
mTOR	Mammalian target of rapamycin
P	Phosphorus
PCoA	Principal coordinate analysis
PERMANOVA	Permutational multivariate ANOVA
PLBP	Porcine lipopolysaccharide binding protein
PZ	Porcine zonulin
ROS	Reactive oxygen species
SCFA	Short chain fatty acid
SEM	Standard error of the means
SID	Standardized ileal digestible
SOD	Superoxide dismutase
TAC	Total antioxidant capacity
Thr	Threonine
TN	Thermos-neutral
TNF-α	Tumor necrosis factor-α
Trp	Tryptophan
TSAA	Total sulfur amino acid
VH	Villus height
VH/CD	Villus height to crypt depth ratio
